# Effect of Processing Treatment and Modified Atmosphere Packing on Carrot’s Microbial Community Structure by Illumina MiSeq Sequencing

**DOI:** 10.3390/molecules27092830

**Published:** 2022-04-29

**Authors:** Katarzyna Ratajczak, Justyna Staninska-Pięta, Jakub Czarny, Paweł Cyplik, Łukasz Wolko, Agnieszka Piotrowska-Cyplik

**Affiliations:** 1 Department of Food Technology of Plant Origin, Poznan University of Life Sciences, Wojska Polskiego 31, 60-624 Poznań, Poland; katarzyna.ratajczak@up.poznan.pl (K.R.); justyna.staninska@up.poznan.pl (J.S.-P.); 2 Institute of Forensic Genetics, Al. Mickiewicza 3/4, 85-071 Bydgoszcz, Poland; pubjc@igs.org.pl; 3 Department of Biotechnology and Food Microbiology, Poznan University of Life Sciences, Wojska Polskiego 48, 60-627 Poznań, Poland; pawel.cyplik@up.poznan.pl; 4 Department of Biochemistry and Biotechnology, Poznan University of Life Sciences, Dojazd 11, 60-632 Poznań, Poland; lukasz.wolko@up.poznan.pl

**Keywords:** microbiome of carrot, modified atmosphere packaging, specific spoilage organisms

## Abstract

The aim of this study was to analyze the microbiome of carrot (*Daucus carota* subsp. *sativus*) subjected to minimal pre-treatment (rinsing in organic acid solution) and packaging in a high-oxygen modified atmosphere, and then stored for 17 days under refrigeration conditions (4 °C). The highest levels of bacteria in the carrot microbiome were characterized, at almost 78%, by bacteria belonging to the Enterobacteriaceae and Pseudomonadaceae families. Rinsing in a solution of ascorbic and citric acids resulted in the improvement of microbiological quality in the first day of storage. However, the use of a high-oxygen modified atmosphere extended the shelf life of the minimally processed product. Compared to carrots stored in air, those stored in high oxygen concentration were characterized by a greater ratio of bacteria belonging to the *Serratia* and *Enterobacter* genera, and a lower ratio belonging to the *Pseudomonas* and *Pantoea* genera. Moreover, the β-biodiversity analysis confirmed that the oxygen concentration was the main factor influencing the differentiation of the metabiomes of the stored carrots. The bacterial strains isolated from carrots identified by molecular methods were mostly pathogenic or potentially pathogenic microorganisms. Neither the minimal pre-treatment nor packaging in high-oxygen atmosphere was able to eliminate the threat of pathogenic bacteria emerging in the product.

## 1. Introduction

Root vegetables make up an essential part of human diet in many regions of the world. Their annual production has been showing a continuous growth trend for two decades. Common carrot is the third most popular vegetable in Europe, and far exceeds the production of other root vegetables. It is characterized by a high content of carbohydrates and fiber, while being low in calories. The annual global production of carrots in 2019 amounted to 44.76 million tons [1].

Approximately 82% of the total carrot yield is sold fresh or minimally processed. Food processing is intended to prepare it for consumption, but it can also extend its shelf life. The most effective method of food preservation is heat treatment. However, it affects the structure of the food product and changes its sensory properties. On the other hand, minimally processed food of plant origin is characterized by sensory characteristics similar to the fresh raw materials and, at the same time, provides the consumer with a product ready for consumption [2].

In order to extend the shelf life of food and ensure its high microbiological quality, non-thermal minimal processing methods are used, which do not change the characteristics of the original food product, but only slightly slow down the growth of undesirable microflora [3,4]. Unfortunately, the use of these methods cannot completely eliminate the risk of an unfavorable change in the microflora composition of food products, which, in turn, accelerates the food spoilage processes [5].

The composition of the carrot metabiome mainly depends on the qualitative and quantitative composition of the soil microorganisms. Natural carrot microflora can be divided into three basic groups: microorganisms that are technologically beneficial or neutral for the inhabited raw material, microorganisms responsible for the processes of raw material spoilage, and pathogenic or potentially pathogenic microorganisms [6]. The first group mainly includes microorganisms added to food as an antimicrobial agent (e.g., producing bacteriocins), and endophytes that are carrot symbiotes (e.g., some strains of *Bacillus subtilis* and mycorrhizal fungi). The remaining specified groups (i.e., spoilage microorganisms and pathogenic and potentially pathogenic microorganisms) constitute the main field of interest, especially in terms of human safety and health [7,8]. Although all microorganisms colonizing the surface of carrots have the ability to decompose plant tissue, a significant number of them remain neutral for the raw material. Unfortunately, root vegetables, such as carrots, have a high risk of contamination with pathogenic microorganisms in the soil [9].

At the stage of cultivation, fertilizers and poor-quality water are the main source of primary microbial contamination of plant material, in addition to soil. During later processing steps, such as harvesting or processing the raw material, carrots are again in contact with microorganisms, including pathogens, and their microflora changes. Secondary infections are caused by non-sterile processing surfaces, machines and tools used in processing in addition to possible cross-contamination between the carrot microflora and the microflora of other food products [10]. Although there are no official requirements for the total number of microorganisms present on fresh vegetables—mainly due to the high content of microorganisms on their surface at the time of harvest—7.0 log CFU/g is widely considered as the use-by limit [11]. Food in which the microflora has developed above this value shows the first signs of deterioration in sensory quality due to the accumulation of secondary metabolites. The effect of the initial low microbiological quality of fresh plant materials such as carrots is their short shelf life. In the case of minimally processed carrots, this period is most often 7 days [11,12].

The microbiological quality of food of plant origin is mainly assessed using classical cultivation methods. However, these methods are not able to detect all microorganisms present in the tested product. Many of these microorganisms are in a state of temporary low metabolic activity or are partially damaged (e.g., by food processing), which means that they do not grow in the laboratory conditions. Others require specific growth conditions that are hard to replicate in a laboratory environment, or overall are too different from standard or enriched media to be cultivable. It should be noted that those microorganisms can also be specific spoilage organisms (SSOs) and pathogens, especially immediately after harvesting or processing the plant material. The use of minimum food processing, appropriately selected for a given plant raw material, mainly in the form of disinfectants, may improve the microbiological quality of food, and thus extend its shelf life [13]. Disinfectants are commonly used in the form of solutions, and have to be approved as safe to use in food processing. Among the most popular disinfectants are solutions of organic acids, because of their antimicrobial properties and minimal effect on the quality of food products [14]. Another popular technique used to prolong the shelf life of food products is packing in modified atmosphere (MAP). It has been noted that high concentration of oxygen in the atmosphere used to pack carrots significantly improved the shelf life of the produce, and also preserved its sensory quality better than low-oxygen MAP [15,16]. However, it is important to know the exact microbiological profile of the raw material in order to properly select the most effective technological treatment and to determine the key points of the processing that determine this profile. The aim of this study is to assess the possibility of using next-generation sequencing as a supplementary method to classic microbiological techniques used to analyze the impact of technological treatments (washing with organic acid solutions and storage in a high-oxygen modified atmosphere) on shaping the carrot metabiome in terms of extending the shelf life and microbiological safety of the product, and assessing the potential of microorganisms belonging to the I risk class for spoilage of carrot.

## 2. Results

### 2.1. Physicochemical Analyzes of Carrot

Physicochemical analyses have shown the effect of the minimal pretreatment (consisting of rinsing in a solution of organic acids—0.5% ascorbic acid and 0.3% citric acid) carried out on the tested carrot in terms of its sensory characteristics (pH, texture and colour) and changes in the composition of the gas atmosphere in the packaging.

#### 2.1.1. Changes in the Concentration of Oxygen and Carbon Dioxide

**Figure 1:**
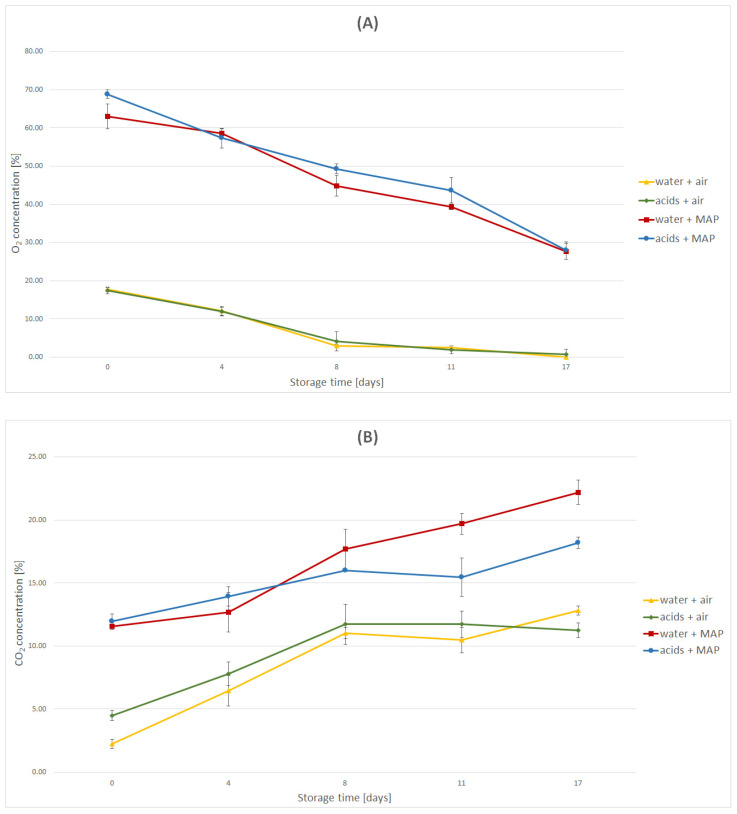

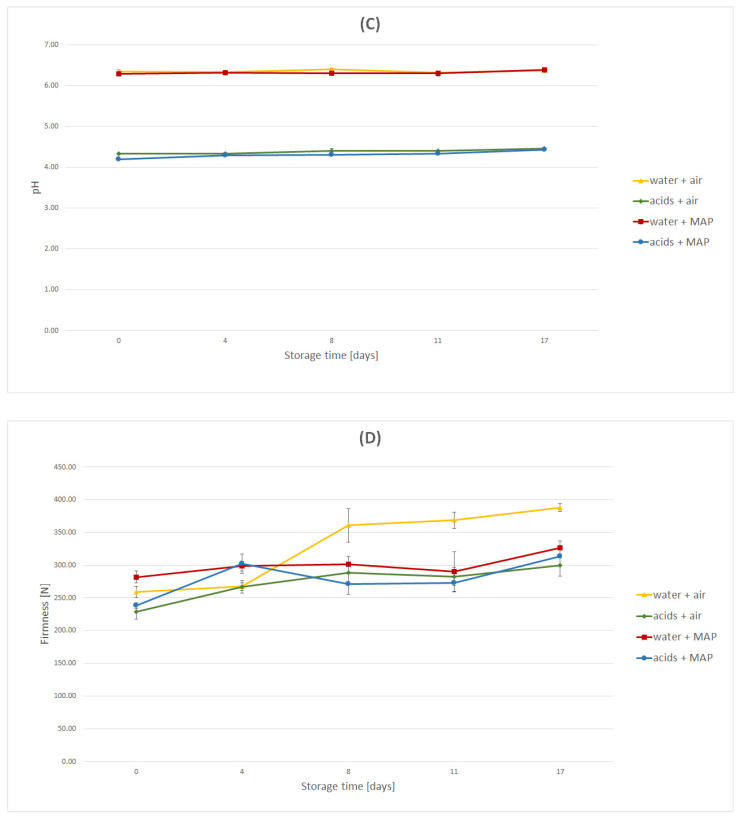

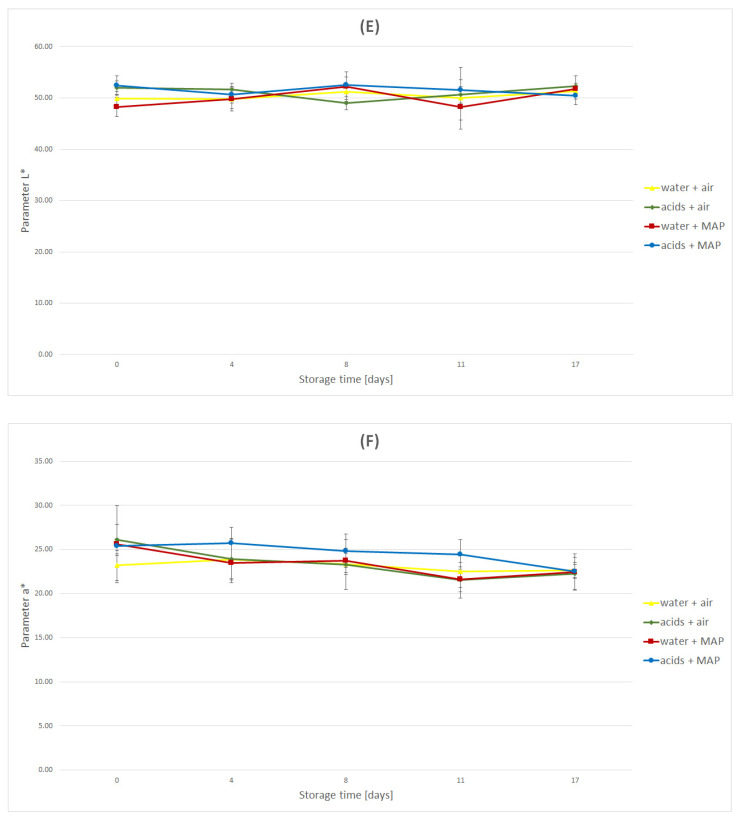

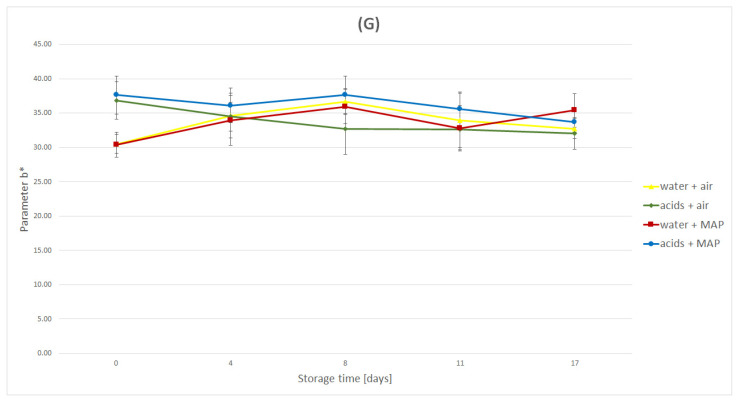
Changes in the concentration of oxygen (**A**), carbon dioxide (**B**), pH (**C**), firmness (**D**), and color (**E**–**G**) of carrots under various packaging and storage conditions.

Natural respiration processes occur in plant tissue, and an increase in the population of microorganisms resulted in the reduction in oxygen content in all variants subjected to rinsing and packaging. In the variants “water + air” and “acids + air”, the greatest reduction in the oxygen content occurred during the first seven days of storing the carrots. On the eleventh day, anaerobic conditions were observed in both variants. In the variants “water + MAP” and “acids + MAP”, the oxygen content decreased evenly over the assumed storage time. Due to the high initial oxygen concentration in the packaging surrounding the carrots, the oxygen was not completely consumed throughout the storage period of the samples. On the seventeenth day, in both variants packed in a modified atmosphere, the oxygen concentration was equal to 27% (Figure 1A). In all packaging variants the carbon dioxide content showed an increasing tendency. In the samples packed in air, the concentration of carbon dioxide increased until the 8th day of the process, after which its value stabilized at the level of 10%. The results obtained for the rinsing variants “water + MAP” and “acids + MAP” indicate a significant impact of the use of an acid solution for rinsing the plant raw material on the reduction in the carbon dioxide content increase in the package. The concentration of carbon dioxide increased in both variants, however, from the eleventh day of storing carrots, a decrease in the upward trend in the variant “acids + MAP” was observed. More importantly, the concentration of carbon dioxide in the “water + MAP” variant exceeded 20%. This level of carbon dioxide is considered potentially toxic to carrots (Figure 1B).

#### 2.1.2. Changes of Carrot’s pH

The pH value of fresh carrot was equal to 6.4. The minimal processing of the plant material significantly influenced the pH values of carrots washed in an organic acid solution, reducing the pH to 4.3. The obtained data show that rinsing in water had no significant effect on the pH of carrots. The pH value did not change significantly during the storage of carrots at refrigerated temperature (Figure 1C).

#### 2.1.3. Changes of Carrot’s Texture

The firmness of raw carrot was equal to 261.75 N. The obtained results indicate a significant influence of the washing solution on the softening of the plant tissue. At the start of the experiment, the firmness of the variants “acids + air” and “acids + MAP” was equal to 228.64 and 238.45 N, while for the variants “water + air” and “water + MAP” the firmness was at 259.17 and 281.87 N, respectively. These results also indicate the beneficial effect of the high-oxygen modified atmosphere on the texture of carrot at the beginning of the experiment. The firmness of carrots in the “water + air” and “water + MAP” variants increased during storage, although a slight decrease in the firmness was noted for the “water + MAP” variant on the eleventh day of the experiment, followed by another increase in firmness until the end of the experiment. However, in the “water + air” variant, the firmness increased significantly between the fourth and eleventh day of the experiment. In the “acids + air” variant, the highest increase in firmness occurred during the first seven days of storage; the subsequent increase in firmness was lower. The texture of carrots in the “acids + MAP” variant was different, as an intensive increase in firmness was noted on the fourth day of storage, followed by a sharp decrease in firmness. The firmness increased again on the seventeenth day of storage. The obtained data indicate that the composition of the atmosphere used to pack carrots influenced the texture of carrots mainly on day zero, however, also during the experiment. The high oxygen content in the packaging in the variants “water + MAP” and “acids + MAP” influenced the firmness of the vegetable tissue. At the same time, the negative effect of organic acid solution on carrots should be highlighted, which was manifested in their softening. On the seventeenth day of the experiment, the highest firmness was noted for carrots from the “water + air” variant (387.86 N), while the firmness of carrots from the remaining three variants of washing and packing was in the range of 299–327 N (Figure 1D).

#### 2.1.4. Changes of Carrot’s Color

Figure 1E–G shows the development of the L*, a* and b* parameters of carrot during storage. The mean values of the L*, a* and b* parameters for raw carrot were 49.53, 22.68 and 31.98, respectively. The minimal processing and packing of carrot did not change the value of the L* parameter. During storage, only slight fluctuations in the L* parameter value were noticed in all tested variants. The greatest variation in the brightness of the product was recorded on the eleventh day, however this phenomenon did not repeat itself on the seventeenth day (Figure 1E). In the case of the value of the a* parameter, the process of rinsing in the organic acid solution resulted in the increase in the parameter value to 26.08 for the “acid + air” variant, and 25.37 for the “acid + MAP” variant. Rinsing in water showed no significant effect on the a* parameter value. The composition of the atmosphere used to pack carrots also did not affect the values of this parameter. During storage, all tested variants showed a slight decreasing tendency for the value of the a* parameter (Figure 1F). Rinsing in organic acids solution significantly influenced the values of the b* parameter. The value of b* parameter on the first day of experiment for carrot variants “acids + air” and “acids + MAP” was, respectively, 36.84 and 37.63, while for variants “water + air” and “water + MAP” it was 30.51 and 30.41. The composition of the atmosphere did not affect the b* parameter value. During the experiment, the b* parameter for the variants “acids + air” and “acids + MAP” showed a downward trend. On the fourth day an increase in the value of the b* parameter for the “acid + MAP” variant was noted. In the “water + air” and “water + MAP” variants, the b* parameter value increased during storage up to the eleventh day. Over the last stage of storage in these variants, the b* parameter value decreased slightly. Importantly, on the seventeenth day, the value of the b* parameter was similar in all variants of carrot (Figure 1G).

### 2.2. Microbiological Analysis

**Figure 2:**
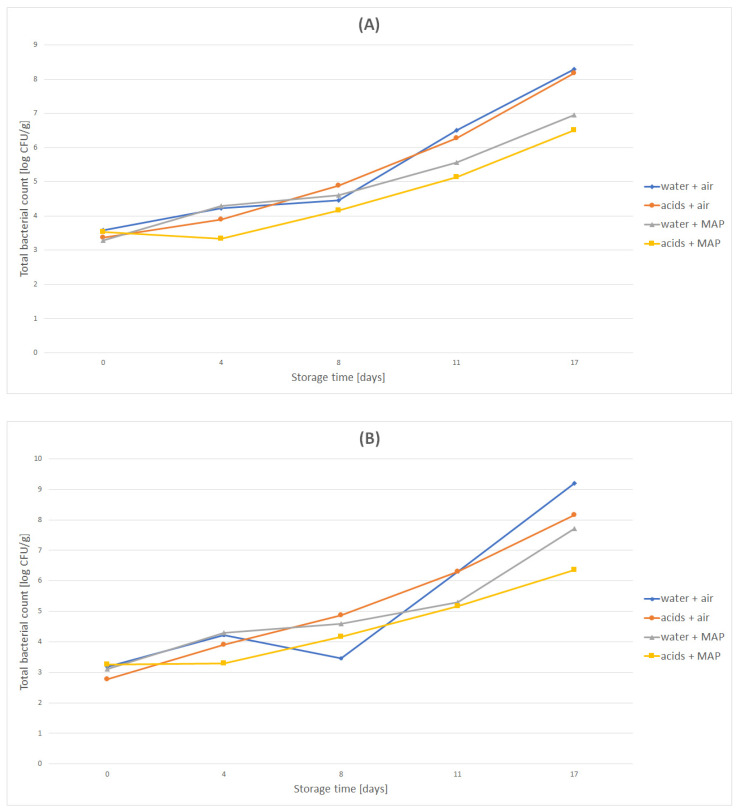

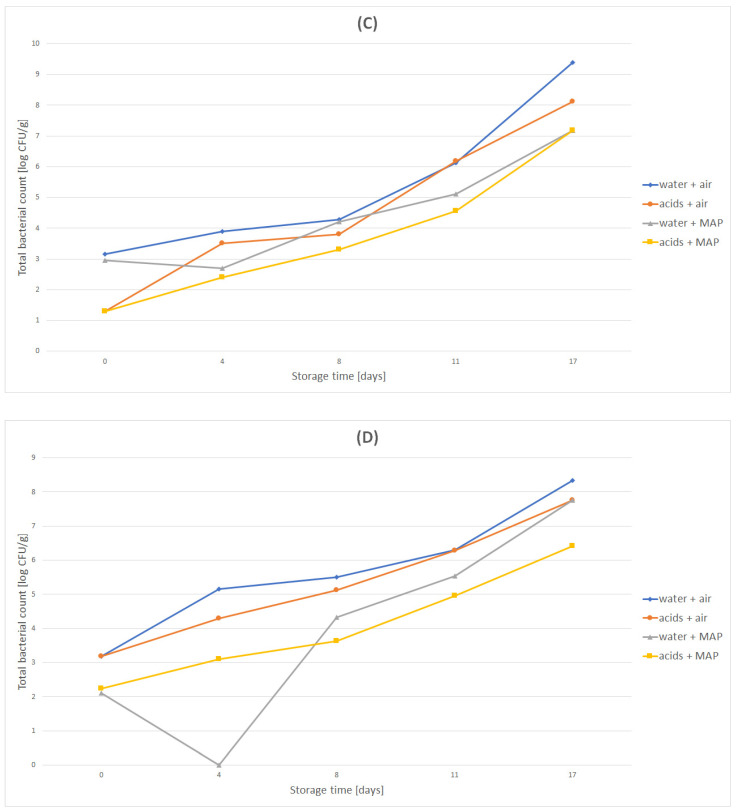
Microbiological quality of minimally processed carrots. (**A**) total number of microorganisms, (**B**) number of Enterobacteriaceae, (**C**) number of coliforms, (**D**) number of *Pseudomonas*.

In fresh carrots, the total number of mesophilic microorganisms was equal to 3.65 log CFU/g, the number of Enterobacteriaceae was equal to 3.25 log CFU/g, the number of coliforms was equal to 3.25 log CFU/g and the number of *Pseudomonas* was equal to 3.2 log CFU/g. Washing the carrots in an organic acid solution as part of minimal processing reduced the number of Enterobacteriaceae and the number of *Pseudomonas*, but did not affect the total number of microorganisms and the number of coliforms. The research showed that the variants of carrots packed in the atmospheric air were characterized by lower microbiological quality during storage. As can be seen in Figure 2A, from the eighth day of storage the total number of microorganisms rapidly increased in the “water + air” variant. In case of carrots packed in a modified atmosphere, a slower increase in the number of microorganisms was observed during the course of the experiment, especially in its second half.

As shown in Figure 2B, values higher by at least one order of magnitude were also observed for the number of Enterobacteriaceae in “water + air” and “acids + air” variants compared to carrots packed in a modified atmosphere.

It was shown that all tested carrot variants, except for the “acids + MAP” variant, were characterized by an intense increase in the value of the number of coliform bacteria already present during the first four days of carrot storage. In the “acids + MAP” variant, the number of coliform bacteria was stable until the eighth day of the experiment. Similar to other microbiological parameters, the carrot variants packed in the air were characterized by the highest number of coliform bacteria (Figure 2C). Their abundance on the final day of the experiment was equal to 9.2 log CFU/g for the “water + air” variant, and 8.16 log CFU/g for the “acids + air” variant. The much lower end values for the number of coliforms for the modified atmosphere packaged variants should be noted. For the “water + MAP” variant, the number of coliforms on the seventeenth day of the experiment was equal to 7.72 log CFU/g, while for the “acids + MAP” variant it was 6.36 log CFU/g.

As shown in Figure 2D, the number of *Pseudomonas* determined in the tested carrots increased in all variants except for the “water + MAP” variant. In this variant, the presence of *Pseudomonas* bacteria was not detected on the fourth day, however this most likely was a technical issue. On the eighth day, an increase in the number of *Pseudomonas* bacteria by four orders of magnitude was noted in this variant. In all tested carrot variants, the increase in the number of *Pseudomonas* bacteria was relatively uniform from day eight onward, but it should be noted that in the last 24 h of the experiment the value of this microbiological indicator in the “water + MAP” variant was similar to the variants of air packed carrot. In the case of the “acids + MAP” variant, the number of *Pseudomonas* bacteria on the final day of the experiment was the lowest among all the examined microbiological indicators and amounted to 6.42 log CFU/g.

### 2.3. Molecular Analysis

#### 2.3.1. NGS Analysis

**Figure 3:**
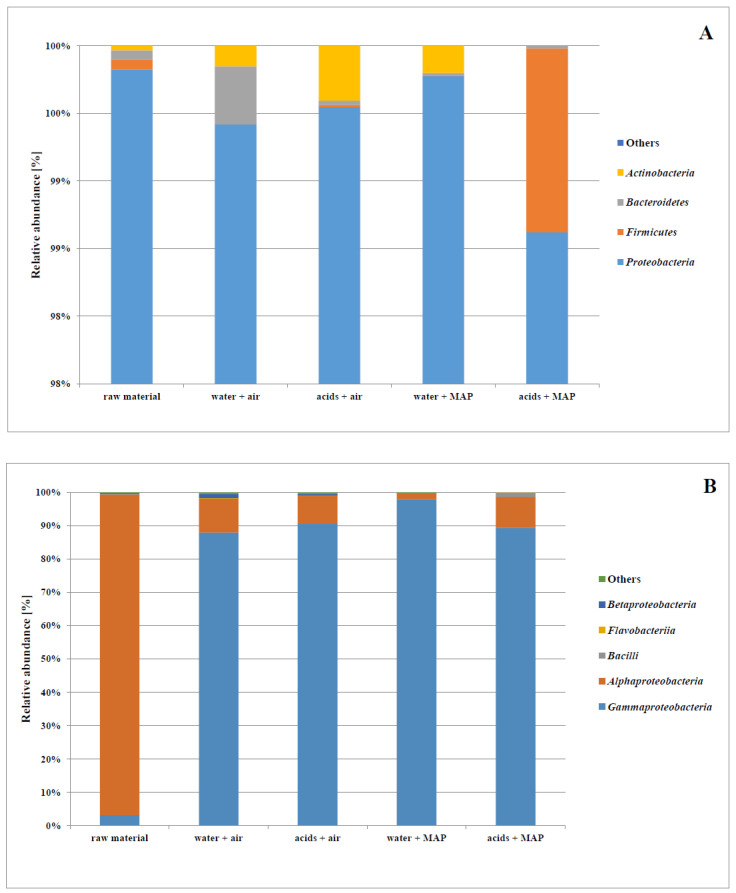

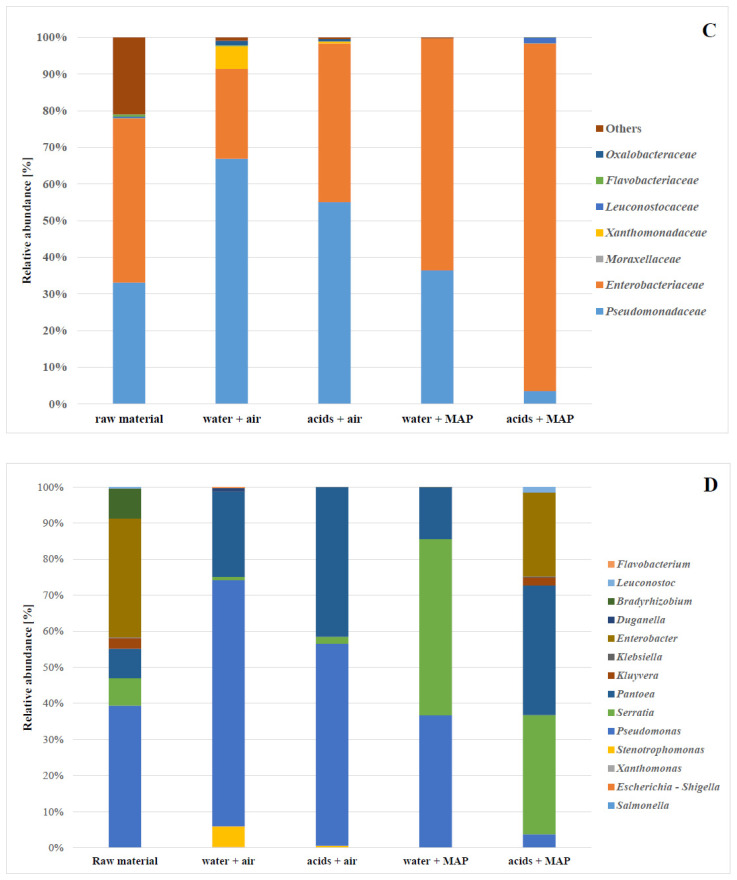
Relative ratio of phyla (**A**), selected classes (**B**), families (**C**), and genera (**D**) of bacteria in raw carrots and minimally processed carrots stored in refrigerated conditions for 17 days.

The analysis with the use of the MiSeq Illumina platform showed that the Proteobacteria phylum dominated in all variants of the tested carrots (Figure 3A). In the case of raw carrots, five main classes were identified, among which the Alphaproteobacteria bacteria were predominant, while the Gammaproteobacteria class, which is the main field of interest in this work, was dominant in the minimally processed carrots (Figure 3B). Using the Illumina MiSeq technology, the authors determined the metapopulation profile of fresh carrots, and then carrots which were rinsed with disinfecting solutions. Sequencing of the 16s region of the rRNA allowed the authors to determine that Proteobacteria and Firmicutes were the dominant phyla in fresh carrots. The dominant class was Gammaproteobacteria, but the authors also identified the presence of the Bacilli, Betaproteobacteria and Alphaproteobacteria classes in the trials.

Many families belong to the Gammaproteobacteria class, however, as can be seen in Figure 3C, the microbiome analysis showed the presence of seven families in the tested plant materials. It was noted that fresh carrots were characterized by the greatest diversity of families from the Gammaproteobacteria class, including bacteria not classified into any of the seven selected families. In all samples of minimally processed raw materials, two families (Enterobacteriaceae and Pseudomonadaceae) were predominant. The relative ratio of these two families in the composition of the microbiota was influenced by the atmosphere used for packaging. The modified atmosphere contributed to a greater ratio of Enterobacteriaceae (65% and 93% for the variants “water + MAP” and “acids + MAP”, respectively). Moreover, in the “acids + MAP” variant, 2% of the identified bacteria were classified to the Leuconostocaceae family, while in the “water + air” variant a 7% ratio of bacteria from the Xanthomonadaceae family was recorded.

The relative ratio of selected genera of bacteria in the carrot microbiome is presented in Figure 3D. It was observed that in fresh carrots the bacteria belonging to the *Pseudomonas* genus accounted for approximately 40% of the total microflora, with the *Enterobacter* genus constituting the second dominant group. In the variants of minimally processed carrots packed in air, the *Pseudomonas* bacteria were the dominant family in the composition of the microflora, while in the variants packed in the modified atmosphere their number was reduced and the Serratia genus was the group of microorganisms with the largest ratio. Moreover, in the “acids + MAP” variant, bacteria of the *Enterobacter* genus accounted for approximately 20%. It can also be noticed that the variants of carrots rinsed in a solution of organic acids were characterized by a higher ratio of bacteria belonging to the *Pantoea* genus. It should also be noted that pathogenic bacteria belonging to the *Salmonella*, *Escherichia* and *Klebsiella* genera have also been identified.

#### 2.3.2. Biodiversity Analysis

The α-biodiversity indicators results are presented in Table 1. For fresh carrot, the indicators were calculated at the level of 47369 readings. An increase in the diversity of the microbial population was observed after seventeen days of storage. It should be noted that packaging in a modified atmosphere influenced the composition of the microbiome and resulted in a reduction in the number of species present in the raw material, as shown by the obtained number of OTUs. The values of the Chao1 index were higher by 30% than the OTU number. The Chao1 index is calculated based on a specific algorithm, and its value does not necessarily result from sequencing errors but from the presence of a significant number of single-read OTUs in the population. The obtained Chao1 values suggest that many species of bacteria with low abundance were identified in the carrot microbiome. The effect of washing in an organic acid solution on the reduction in the number of species was also observed. The values of the Shannon function also indicated an increase in the diversity of metabiomes in the samples of minimally processed carrots, although there was no clear trend of the effect of rinsing in the organic acid solution on its basis. However, it was possible to conclude that the high-oxygen modified atmosphere decreased the species richness of the carrot metabiome. Analysis of biodiversity indicated that the Simpson index increased with the storage of minimally processed carrots, which was associated with an increase in the number of OTUs and resulted in less noticeable dominance of some species. More importantly, in the case of this index it was also observed that the minimal pre-treatment and packaging in the modified atmosphere decreased its value.

**Figure 4:**
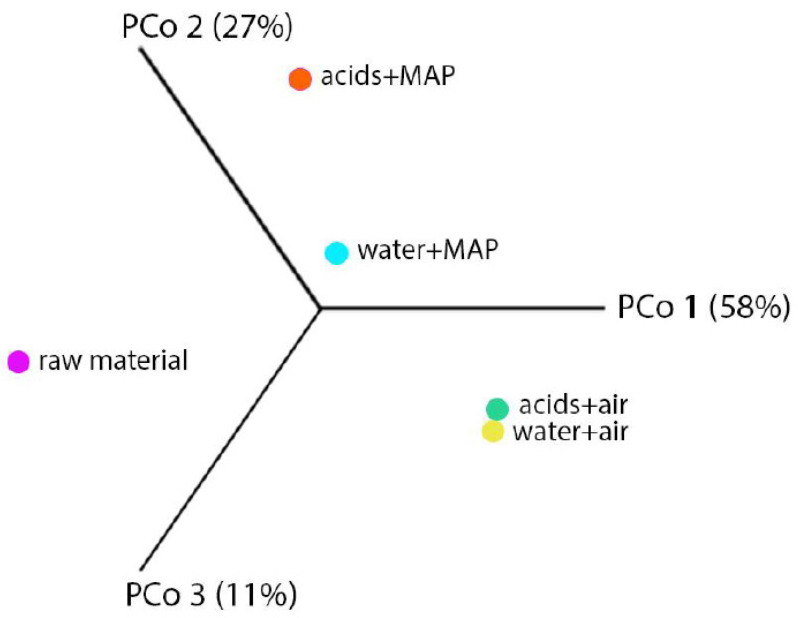
Principal coordinates analysis (PCoA) based on Bray–Curtis index for carrot microbiome stored under refrigerated conditions.

The β-biodiversity of carrot was determined by principal component analysis (PCoA) (Figure 4). The main components of PCo1, PCo2 and PCo3 were determined as combinations of the studied variables and represented the percentage differences (38%, 18% and 16%, respectively) between the analyzed carrot variants. The components PCo1, PCo2 and PCo3 represented the differences in the biodiversity of the microbiomes in 58%, 27% and 11%, respectively. As can be seen in Figure 4, the microbiome of fresh carrot differed from that of minimally processed carrots after 17 days of storage. Interestingly, this analysis also shows that the atmosphere used for packaging was the main factor influencing the biodiversity of carrot microflora.

#### 2.3.3. Identification of Dominant Strains in Carrot Microflora

The use of first-generation sequencing using the Sanger method allowed for the identification of selected strains isolated from minimally processed carrot with a probability of at least 96%. The results of the identification, alongside the agars from which the original colonies were recovered from, are presented in Table 2. Strains marked as MK were isolated from carrot subjected to washing in an organic acid solution and then packed in a modified atmosphere, while the strains marked with the abbreviation MW were isolated from ordinary carrot subjected to washing in water and then packing in a modified atmosphere.

Among the identified isolates, attention should be paid to both repeating strains, particularly *Klebsiella*, which is a pathogenic bacteria, and *Enterobacter tabaci YIM Hb-3,* which is a phytopathogen. Among the identified microorganisms, the strain of *Salmonella enterica* was originally recovered from CFC agar (used to determine the number of *Pseudomonas bacteria*). More importantly, this strain was not detected by a standard culture method used for the determination of the presence of *Salmonella*. It should also be noted that the presence of *Salmonella* was also confirmed using the NGS method in the previous experimental step. Moreover, it should be emphasized that all of the identified strains (except for *E. tabaci*) were classified as pathogenic or potentially pathogenic for humans.

## 3. Discussion

### 3.1. Physicochemical Analyses

In this study the oxygen content in the variants packed in the modified atmosphere was 27% after 17 days of storage. Ayhan et al., who conducted an experiment on carrot rings treated with a citric acid solution and packed in a high-oxygen modified atmosphere, reported the total oxygen consumption on the fourteenth day of the experiment [15]. However, it is worth noting that unshredded carrots used the oxygen from the surrounding atmosphere more slowly, as evidenced by Leceta et al. [17]. Shredding of the carrot in this study affected the speed of oxygen consumption, however the use of a high-oxygen modified atmosphere prevented the occurrence of anaerobic conditions. The food industry mostly relies on low-oxygen and high-carbon dioxide atmospheres to promote conditions in which many of the aerobic spoilage microorganisms cannot grow [18]. However, it has been proven that high-oxygen atmosphere inhibits the growth of spoilage microorganisms while preventing undesirable changes in sensory qualities of plant-based products [19], which supports the results obtained within this study.

In this experiment, the pH of the carrot was not affected either by the atmosphere used for packing, nor by storage itself. Rinsing in the solution of ascorbic and citric acids had a significant effect on the pH of product, lowering it from an initial 6.4 to 4.3. The effect of ascorbic acid solution lowering the pH value of shredded carrots rinsed with it was confirmed by Xylia et al. [20]. Authors also noted that pH value decreased during the storage of carrots, however this could be due to the type of anaerobic storage used. In turn, Alegria et al. confirmed that rinsing in water, chlorine solution or ozonated water had little effect on the pH of shredded carrots [21]. In a later experiment, Alegria et al. noted that the pH of shredded carrots washed in a disinfecting solution did not change during seven days of storage [12]. Additionally, Ayhan et al. found no changes in the pH value of carrots packed in a modified atmosphere stored for fifteen days under refrigeration [15].

The obtained results of carrot texture analysis indicated that rinsing in acid solution and high oxygen concentration in the atmosphere resulted in weaker hardening of carrot tissues. Interestingly, the experiment carried out by Leceta et al. showed that carrot tissues packed in a modified atmosphere with a low oxygen content softened during storage [17]. However, the results obtained by Klaiber et al. were consistent with the results obtained in this study and indicated the hardening of ground carrot during storage [22]. Carrot hardening is a natural process resulting from the dehydration of tissues combined with their lignification. According to Ayhan et al., no significant changes in the texture of the raw material were observed during the fourteen days of storing minimally processed carrots [15], which differed from results obtained in this work. Changes in texture are generally unwanted in the fresh-like minimally processed products, and it has to be noted that the obtained results prove that a high-oxygen modified atmosphere is the main positive factor allowing the carrot to retain its fresh-like qualities.

An experiment conducted by Leceta et al. found that storing carrots in a modified atmosphere affected the brightness of carrots, observed as an increase in the L* parameter value [17]. The obtained values of the L*, a* and b* parameters were similar to the values obtained in the paper below, although they pointed to overall brighter carrots. It should be noted that the grinding of carrots in this study could have had an impact on the color reading by creating shadow zones between its particles. Klaiber et al. also did not observe any changes in the color of carrots during their storage for nine days [22]. In turn, Chauhan et al. observed that the use of minimal pretreatment (ozonation and modified atmosphere packaging) stabilized the color of carrots [23]. The darkening of carrot tissue is associated with progressive lignification and enzymatic browning of the produce. This suggests that minimal pre-treatment may prevent adverse sensory changes.

### 3.2. Microbiological Analyses

It should be noted that the use of an organic acid solution for rinsing of carrot as part of the minimal pre-treatment resulted in the significant reduction in the number of specific microorganisms present on the carrots, mainly Enterobacteriaceae bacteria. However, during the storage of minimally processed carrots, the high oxygen content in the modified atmosphere was the main factor which slowed down the development of a significant part of the microflora. Most of the microorganisms responsible for the deterioration of plant tissues are either anaerobic or facultative anaerobic, and high levels of oxygen could be toxic even on aerobic bacteria such as *Pseudomonas*. Results of the experiment conducted by Baez et al. confirm such hypothesis and show that the use of hyperbaric oxygen (100% concentration) inhibited the growth of *Escherichia coli*, *Bacillus subtilis* and *Enterococcus faecalis*, which correlates with results obtained in this study [24]. The total microbial count in fresh carrot analyzed in this study was equal to 3.65 log CFU/g. A different experiment, conducted on fresh carrots by Määttä et al. showed that the total number of mesophilic bacteria was higher by two logarithmic cycles when compared to the results obtained in this research [25]. Additionally, Leceta et al. [17] and Fiedler et al. [26] reported that the total number of mesophilic microorganisms present on carrots amounted to at least 5.5 log CFU/g. It turns out that the total mesophilic bacteria count observed on fresh carrot in this study classifies the tested carrot as a raw material with good initial microbiological quality. Further results of the microbiological analysis obtained in this study correspond well with the research of Määttä et al., in which the authors reported the presence of coliforms and Enterobacteriaceae on carrots at the level of 4.0 and 3.0 log CFU/g, respectively [25]. Määttä et al. also found that washing the carrots before processing resulted in a reduction in the number of bacteria from these two groups, which is consistent with the results obtained in this study. Fiedler et al. established that the Enterobacteriaceae bacteria are present in commercially available minimally processed carrots at an abundance higher by two orders of magnitude than the results obtained in this study [26]. An experiment conducted by Chauhan et al. also confirmed that the use of a modified atmosphere for packing shredded carrots allowed to slow down the growth of the total number of microorganisms and coliform bacteria [23]. Similar results were obtained by Leceta et al., as the minimal treatment significantly reduced the total number of bacteria, the number of *Pseudomonas* and *Bacillus*, and it should be noted that the tested carrots had microbiological indexes at the level of 5.0 log CFU/g [17]. In turn, the results obtained by Hidemi et al. confirmed that the dominant microorganisms on the surface of carrots were *Pseudomonas*, *Enterobacter* and *Pantoea*, and the authors also detected the presence of lactic acid bacteria [27].

### 3.3. NGS and Sanger Sequencing Analyses

This composition of bacterial classes within the Proteobacteria phylum observed in this study corresponds well with the results obtained by Dharmarha et al. [6], who determined that 78% of all identified bacteria belong to the Pseudomonadaceae, Enterobacteriaceae, Oxalobacteraceae, Bacillaceae or Paenibacillaceae families. Different results from those obtained in this study were noted by Lampert et al. during the comparison of the impact of a high concentration of carbon dioxide CO_2_ on the formation of the carrot metabiome [28]. Lampert et al. observed that carrots stored in the air were dominated by the Rickettsiaceae families, and, to a lesser extent, by the Pseudomonadaceae, Actinomycetaceae and Rhizobiaceae. The Enterobacteriaceae, Pseudomonadaceae and Lactobacillaceae families dominated in carrots stored in 98% of CO_2_, which partially corresponds to the results obtained in this study. Bacteria from these families are relatively anaerobic microorganisms, and their development was undoubtedly related to the composition of the atmosphere. More importantly, three isolates collected from carrots were identified by Lampert et al. as *Pantoea agglomarens*, *Rahnella aquatilis* and *Leuconostoc mesenteroides* [28]. No lactic acid bacteria isolates were identified in the framework of this study, and most of the 14 isolated and identified strains belonged to the Enterobacteriaceae family. This difference between the results obtained in this research and the results found in the literature may be due to the primary agar from which the colonies were recovered for later identification.

Lampert et al. also confirmed the existence of differences between the diversity of the metabiome of carrots packed in air and that of carrots packed in CO_2_. Interestingly, these authors also determined that carrots stored in the air exhibited higher OTU numbers and higher Shannon function values. Moreover, the values of the Shannon function were similar to those obtained in this study, but the number of OTUs for the population of carrots studied was significantly higher [28]. Additionally, Dharmarha et al. confirmed that the disinfectants used in their experiment influenced the composition of the carrot metabiome, including its biodiversity [6].

## 4. Materials and Methods

### 4.1. Sample Procurement

The analyses were carried out using carrots of the Bangor variety obtained from conventional cultivation in the vicinity of Kórnik (Greater Poland Voivodeship, Poland). The carrots were harvested in September 2016.

### 4.2. Preparation of Carrots

The carrots were pre-washed with tap water, then hand peeled, washed again and blotted dry. Tap water was used to wash the carrots in all steps of preparation. The raw material was then shredded using a Robot Coupe CL 50 Ultra (Vincennes). The shredded carrots were rinsed with running water or with a rinsing solution consisting of a mixture of 0.5% ascorbic acid (POCH) and 0.3% citric acid (POCH) for 5 min at the ratio of 1 kg of carrots per 5 L. The concentration of organic acids was selected after literature research due to its minimal effect on carrot tissue and sensoric qualities of the final product. After rinsing in an organic acid solution and running water, the shredded carrots were dried using a manual vegetable centrifuge (Zepter, Switzerland). The raw material prepared this way was packed using a Multivac T-1200 (Wolfertschwenden, Germany) packaging machine with a WITT KM 100/200-3 MEM gas mixer (Witt-Gasetechnik, Germany) in atmospheric air or in a modified atmosphere (70% O_2_ + 20% N_2_ + 10% CO_2_). The carrots were packed in the amount of 150 g in polypropylene trays (205 mm length, 160 mm width, 60 mm height) and closed with a polyolefin foil (oxygen permeability equal to 3000 cm^3^/m^2^/24 h) at a relative humidity of 85%. The packaged carrot variants were stored at 4 °C for 17 days.

### 4.3. Physicochemical Analyzes of Carrot

#### 4.3.1. Assessment of Gas Content Changes in the Packaging

The gas content was measured using an OXYBABY gas analyzer (Witt-Gasetechnik, Germany). The measurements were carried out by puncturing a polyolefin film secured with a rubber seal with a measuring needle. Measurements were conducted in triplicate for each sample.

#### 4.3.2. Determination of Carrot pH Change

The pH of the carrots was measured using a CP-401 pH meter (Elmetron, Poland). The pH meter electrode was placed in 30 g of the carrots shredded as described above. Measurements were conducted in triplicate for each sample.

#### 4.3.3. Evaluation of Carrot Texture Change

Carrot texture was examined using a TA.XT.plus Texture Analyzer (Stable Micro Systems, Godalming, UK) with a 50 kg head. A 10 g sample of carrots was placed in a Kramer five-blade chamber (HDP/KS5) and subjected to shear using the Exponent 6.1.14 (Stable Micro Systems) built-in the program operating the texturometer for the Kramer chamber test. Test conditions: shear force 50 kg, test speed 1.5 mm/s, speed after measurement 10 mm/s, distance 39 mm.

#### 4.3.4. Evaluation of Carrot Color Change

The color of the carrots was measured in the CIELab color space (L*, a*, b*) using a Chroma Meter CR-400 colorimeter (Konica Minolta, Quarry Bay, Hong Kong). Carrots were arranged into an opaque layer for measurement. Measurements were made in triplicate for each trial.

### 4.4. Microbiological Analyzes

Determination of microbiological quality was carried out using standard method in accordance with ISO standards. The measurements were conducted for the total number of microorganisms (medium used was standard agar with glucose and nutrient broth), the number of Enterobacteriaceae (medium used was VRBG agar), the number of coliforms (medium used was MacConkey agar) and the number of *Pseudomonas* (medium used was CFC agar with *Pseudomonas* CFC Supplement added). In order to prepare the samples, 10 g of carrots were homogenized with 90 mL of physiological saline using a Stomacher Bag Mixer^®^ 400 machine (Interscience, Saint Nom la Brétèche, France) for 3 min, and decimal dilutions were prepared using the obtained solution. All inoculations were carried using the pour plate procedure.

### 4.5. Molecular Analyzes

#### 4.5.1. NGS Analysis

##### DNA Extraction

Samples of 5 g were collected from fresh, untreated carrots and from minimally processed carrots after 17 days of storage. Samples were shaken with 5 mL of saline for 10 min. Total bacterial DNA was isolated from 1.5 mL of the resulting solution using the Genomic Mini Kit (A&A Biotechnology, Gdańsk, Poland). The amount of DNA was determined using the Qubit dsDNA HS Assay Kit (Thermo Fisher Scientific, Waltham, MA, USA.) with a Qubit 3.0 fluorimeter (Thermo Fisher Scientific). The purity of the samples was checked on 5 μL of isolated DNA using E-Gel iBase (Invitrogen, Waltham, MA, USA). and a 0.8% agarose gel.

##### PCR Amplification

Universal primers 515F and 806R were used to amplify the IV region of the bacterial 16s rRNA [27]. The PCR reaction mixture contained: 1× PCR reaction mix, 100 ng of genomic DNA, 0.25 µL of each primer and 5U Taq Polymerase (A&A Biotechnology). PCR amplification was performed using the following conditions: initial denaturation at 95 °C for 3 min; 35 cycles of denaturation at 94 °C for 30 s, annealing of the primers at 52 °C for 30 s and 72 °C for 2 min, then final extension at 72 °C for 10 min. The PCR products were purified using a Clean-Up column (A&A Biotechnology) according to the manufacturer’s protocol.

##### 16S rRNA Gene Sequencing

Libraries were constructed based on a re-amplification reaction using the Illumina system fusion primers under conditions identical to those used in the first PCR reaction, using the sequencing primers shown in Table 3 [29]. The PCR products were re-purified using a Clean-Up column (A&A Biotechnology). The DNA concentration was determined using the Qubit dsDNA HS Assay Kit and the Qubit 3.0 fluorimeter (Thermo Fisher Scientific). Libraries were denatured with a 0.2 N NaOH solution then diluted with a HT1 buffer (Illumina, San Diego, CA, USA) to a final concentration of 8 pM, and a PhiX control (Illumina) was added to achieve a final concentration of 40%. Sequencing was performed using an Illumina MiSeq sequencer (Illumina) with paired ends (2 × 250) of the MiSeq Reagent Kits v2 (Illumina). The sequencing datasets generated and analyzed during the current study are available in the SRA repository, under the identifier BioProject PRJNA664904.

##### Bioinformatic Analysis

CLC Genomic Workbench 8.5 and CLC Microbial Genomics Module 1.2 (Qiagen, Germantown, MD, USA) were used to interpret and process the obtained data. After sequencing, all readings were demultiplexed based on the sequence of indexes per sample. A complementary match of the sequencing reads from both ends was then performed (70% of all readings) and the obtained fragments were cut to a length of 291 nt. Only the fragments obtained by complementary matching and cutting were used for further analyses. Chimeric sequences were also separated from the reading pool. The sequences prepared this way were assigned to operational taxonomic units (OTU). The readings were then compared with the SILVA v119 database on the basis of 97% sequence similarity of the 16s rRNA genes [30]. Calculation of α-biodiversity indicators such as: OTU number, Chao1 index, Shannon function and Simpson index were performed according to instructions provided by [31].

#### 4.5.2. Sanger Sequencing of Selected Strains Isolated from Carrot

##### Strain Selection and Isolation

Selected colonies were selected randomly and collected from the Petri dishes with a sterile loop. Colonies were taken from following agars: CFC, M-17, McConkey, MRS, MYP, and VRBG. Reductive inoculation was then performed on nutrient agar with glucose. After incubation at 37 °C for 24 h, the reductive inoculation was repeated to purify the harvested strain. After another incubation at 37 °C for 24 h, a single colony was used for inoculation of a 10 mL test tube containing nutrient broth with glucose. The broth was incubated at 37 °C for 24 h with constant shaking. An amount of 1 mL of the bacterial culture was protected with 0.5 mL glycerol (BTL) and stored at −80°C. A total of 14 isolates were obtained.

##### DNA Extraction

Total DNA was isolated from 1 mL of bacterial culture using the Genomic Mini Kit (A&A Biotechnology) according to the manufacturer’s protocol. The amount of DNA was determined using the Qubit dsDNA HS Assay Kit (Thermo Fisher Scientific) on a Qubit 3.0 fluorimeter (Thermo Fisher Scientific) according to the manufacturer’s protocol.

##### PCR Amplification and Sequencing

Amplification and sequencing of the 16s rRNA gene was performed according to the methodology used by [32]. Primers Epsilon F and 1510R (Invitrogen) were used to amplify the 16s rRNA region. The reaction mixture consisted of 10 µL of PCR buffer (Invitrogen), 8 µL of the nucleotide mix (Invitrogen), 2 μL of sample DNA (at a concentration of 120 ng/mL), 0.2 μL of Taq polymerase (Invitrogen) and 2.5 μL of each primer. The mixture was topped to 100 μL with PCR water. The PCR reaction was performed under the following conditions: initial denaturation at 95 °C (5 min), then 30 cycles of denaturation at 95°C (1 min), annealing at 55 °C (1 min) and elongation at 72 °C (1 min). The last step was final elongation at 72 °C, lasting 10 min. The PCR products were purified with a Clean-Up column (A&A Biotechnology) according to the manufacturer’s protocol.

A 16s rRNA coding region sequencing was performed using primers previously used for the PCR reaction. The sequencing reaction was performed using the BigDye Terminator v1.1 Cycle Sequencing Kit (Thermo Fisher Scientific). The reaction mixture consisted of 4 µL of Ready Reaction Mix, 2 µL of BigDye sequence buffer, 4 µL of primers, and 2 µL of template (PCR product). The mixture was topped to 20 μL with PCR water. The reaction was performed under the following conditions: initial denaturation at 96 °C (1 min), 25 cycles of denaturation at 96 °C (10 s), annealing at 50 °C (5 s) and extension at 60 °C (4 min). Reaction products were purified using the BigDye Terminator PCR Purification Kit (Thermo Fisher Scientific) according to the manufacturer’s protocol. The sequencing products were then separated by denaturing electrophoresis on a 0.2 mm 4% polyacrylamide gel. Reading was performed using an ABI 377 DNA Sequencer (Applied Biosystems). The final sequencing results were recorded using the ABI Prism 377 program. Identification of the microorganisms was performed by comparing the obtained sequences with the GenBank database [32].

### 4.6. Statistical Analysis

Statistical processing of the obtained results was performed using the Shapiro–Wilk test and the analysis of the uniformity of variance in addition to the Kruskal–Wallis test using the Statistica 13 program (StatSoft).

## 5. Conclusions

The use of a specific minimum technological food processing, appropriately selected for a given low-processed plant raw material, mainly in the form of organic disinfectants, may improve the microbiological quality of food and thus extend its shelf life. However, as shown by the conducted research, it is very important to know the exact microbiological profile of the raw material in order to properly select the most effective technological treatment and indicate the key points of the processing that determine the microbiological profile of a given product.

The carried-out analysis of the carrot metabiome allowed the precise determination of the influence of the technological measures applied on the direction of changes in the carrot microbiome, which affected the quality and durability of the stored food. The applied technological processes—consisting of washing the raw material with water or a mix of ascorbic and citric acids solutions, and their storage in air or in modified atmosphere—significantly changed the original composition of the carrot metabiome, with bacteria belonging to the Eneterobacteriaceae and Pseudomonadaceae families always being the dominant microorganisms. It should, therefore, be concluded that these groups of bacteria are mainly responsible for the spoilage of carrots. These observations were partially confirmed by the species analysis of the randomized strains isolated from the stored carrots—most of those strains belonged to the Enterobacteriaceae family. It should be emphasized that excessive prolongation of the shelf life of low-processed food under specific, intentionally created storage conditions may lead to the stimulation of the development of potentially pathogenic groups of microorganisms not only responsible for spoilage of food, but also threatening human health. At the same time, it has to be noted that minimal treatment technologies allowed the product to retain its sensory values better but were also unable to eliminate the threat of the growth of pathogenic or potentially pathogenic bacteria. Still, it seems that high-oxygen modified atmosphere may find a use in the development of a minimally processed plant-based product with a prolonged shelf life and prolonged fresh-like sensory qualities, as proven by the comparison between carrot packed in high-oxygen modified atmosphere and packing in air. Organic acids, which have GRAS safety status, may be a good solution for a low-cost technology that allows the improvement of the microbiological quality of fresh-like plant-based foods, however only in the short-term.

## Figures and Tables

**Table 1:** Values of α-biodiversity indicators of the carrot metabiome.

Sample	OTU Number	Chao1 Index	Shannon Function	Simpson Index
Raw carrot	96	129	3.03	0.81
After 17 days of storage				
Water + air	278	293	5.52	0.96
Acids + air	254	268	4.81	0.93
Water + MAP	201	231	4.01	0.89
Acids + MAP	179	200	4.39	0.92

**Table 2:** Bacterial isolates recovered from carrot.

Isolate	Taxon	Source Agar
MK1	*Klebsiella quasipneumoniae* subsp. *quasipneumoniae 01A030*	McConkey
MK2	*Klebsiella pneumoniae NF21*	CFC
MK3	*Salmonella enterica* subsp. *enterica serovar Enteritidis SA28*	CFC
MK4	*Enterobacter tabaci YIM Hb-3*	M-17
MK5	*Enterobacter kobei JCM 8580*	MRS
MK6	*Enterobacter cancerogenus T2P18*	VRBG
MK7	*Citrobacter freundii SY-CF14*	VRBG
MK8	*Enterobacter tabaci YIM Hb-3*	MRS
MK9	*Klebsiella quasipneumoniae* subsp. *quasipneumoniae 01A030*	MYP
MW1	*Enterobacter tabaci YIM Hb-3*	McConkey
MW2	*Enterobacter tabaci YIM Hb-3*	CFC
MW3	*Enterobacter asburiae CEES15*	M-17
MW4	*Klebsiella pneumoniae Ma19*	VRBG
MW5	*Enterobacter tabaci YIM Hb-3*	MYP

**Table 3:** Primers used in the amplification reaction and for sequencing [29].

Primers	Sequence (od 5′ do 3′)
PCR amplification	
Forward 515F	AATGATACGGCGACCACCGAGATCTACACTATGGTAATTGTGTGCCAGCMGCCGCGGTAA ^1^
Reverse 806R	CAAGCAGAAGACGGCATACGAGATXXXXXXAGTCAGTCAGCCGGACTACHVGGGTWTCTAAT ^2^
**Sequencing**	
Read1	(TATGGTAATT) ^6a^ (GT) ^7a^ (GTGCCAGCMGCCGCGGTAA) ^8a^
Read2	(AGTCAGTCAG) ^6b^ (CC) ^7b^ (GGACTACHVGGGTWTCTAAT) ^8b^
Index	(ATTAGAWACCCBDGTAGTCC) ^6c^ (GG) ^7c^ (CTGACTGACT) ^8c^

^1^—contains 5′ end adapters of the Illumina system, forward primer attachment region, forward primer linker and forward primer sequence; ^2^—contains 3′end adapters of the Illumina system, “golay barcode” sequence label, reverse primer attachment region, reverse primer linker and reverse starter; ^6a^—forward primer attachment region; ^6b^—reverse primer attachment region; ^6c^—segment complementary to reverse primer; ^7a^—forward primer linker; ^7b^—reverse primer linker; ^7c^—segment complementary to reverse primer linker; ^8a^—forward primer; ^8b^—reverse primer; ^8c^—segment complementary to the reverse primer attachment region.

## Data Availability

Not applicable.

## Sample Availability

Samples of the compounds are not available from the authors.
